# Algorithmic management and employees’ work withdrawal behavior: the roles of AI anxiety and perceived algorithmic transparency

**DOI:** 10.3389/fpsyg.2026.1916936

**Published:** 2026-10-21

**Authors:** Yong-hui Gao, Yu-feng Pan

**Affiliations:** School of Management, Northwest Minzu University, Lanzhou, China

**Keywords:** AI anxiety, algorithmic management, perceived algorithmic transparency, technostress theory, work withdrawal behavior

## Abstract

**Introduction:**

As artificial intelligence and algorithmic systems are increasingly incorporated into organizational management, scholarly attention has been directed toward employees’ psychological and behavioral responses. Drawing on technostress theory, this study examines the association between algorithmic management and employees’ work withdrawal behavior, focusing on the indirect association through AI anxiety and the moderating role of perceived algorithmic transparency.

**Methods:**

A cross-sectional questionnaire survey was conducted among corporate employees in China, yielding a final analytical sample of 346 employees. The proposed relationships were examined using statistical analyses of direct, indirect, and moderating associations.

**Results:**

Algorithmic management was positively associated with work withdrawal behavior and AI anxiety. AI anxiety was also positively associated with work withdrawal behavior, and bootstrap analyses indicated a statistically significant indirect association through AI anxiety. Furthermore, perceived algorithmic transparency negatively moderated the association between algorithmic management and AI anxiety. The conditional indirect association through AI anxiety was weaker at higher levels of perceived algorithmic transparency.

**Discussion:**

These findings are consistent with the proposed moderated mediation model and highlight AI anxiety and perceived algorithmic transparency as potentially relevant factors in understanding employees’ responses to algorithmic management. However, the cross-sectional design does not establish temporal precedence or causal relationships.

## 1 Introduction

With the rapid development of the digital economy and artificial intelligence technologies, organizational management is undergoing profound transformation. Big data, artificial intelligence, machine learning, and automated decision-making systems are increasingly being applied to management activities such as task allocation, performance evaluation, behavioral monitoring, work scheduling, and reward-and-penalty feedback, giving rise to a pronounced trend toward the digitalization and algorithmization of organizational management (Kellogg et al., 2020; Lee et al., 2015; Rosenblat and Stark, 2015). To some extent, the introduction of algorithmic systems has improved organizational efficiency and managerial precision; however, it has also reshaped employees’ work environment and experiences. In the workplace, employees are no longer confronted solely with supervision and evaluation from traditional managers but must also continuously adapt to data tracking, real-time feedback, and automated decision-making enabled by algorithmic systems. Consequently, employees’ psychological states and behavioral responses to algorithmic management have gradually become significant issues in organizational behavior research.

Against the backdrop of deepening digital management, employees’ work withdrawal behavior deserves particular attention. Work withdrawal behavior refers to employees’ voluntary reduction of their connection with work when confronted with job-related stress, dissatisfaction, or threats. It is typically manifested in psychological disengagement, reduced work investment, diminished proactive behavior, avoidance of work tasks, tardiness, early departure, or intentions to leave the workplace (Hanisch and Hulin, 1990; Lehman and Simpson, 1992). As a typical form of negative workplace behavior, work withdrawal not only undermines individual job performance and career development but also weakens team collaboration efficiency, increases organizational management costs, and ultimately impairs overall organizational functioning (Berry et al., 2012; Hom and Kinicki, 2001). Therefore, exploring the antecedents of employees’ work withdrawal behavior is important for understanding negative behavioral responses in digitally managed work environments.

Existing studies have extensively examined the antecedents of employees’ work withdrawal behavior, focusing primarily on factors such as job stress, organizational justice, psychological contract breach, job burnout, leadership style, and organizational support (Chiu and Francesco, 2003; Podsakoff et al., 2007). These studies provide an important foundation for explaining why employees reduce work engagement, avoid job tasks, and develop psychological detachment from work. However, as algorithmic systems have increasingly entered organizational management processes, employees’ managerial context has also changed. Compared with traditional management approaches, algorithmic management is characterized by real-time operations, continuity, datafication, and automation. Employees’ work processes, behavioral performance, and performance outcomes can be continuously recorded, analyzed, and evaluated, thereby influencing task assignments, performance appraisals, and organizational rewards and punishments (Parent-Rocheleau and Parker, 2022; Wood et al., 2019). Accordingly, whether the explanatory frameworks of work withdrawal developed in traditional management settings can adequately account for withdrawal behavior under algorithmic management remains an issue that warrants further research.

As an increasingly prominent managerial approach, algorithmic management may reshape employees’ perceptions of job control, performance evaluation, and career security; however, its consequences are not uniformly negative (Deng et al., 2025; Zhu et al., 2024). Depending on their function, design, implementation, organizational context, and level of human oversight, algorithmic systems may provide useful resources by improving the coordination, scheduling efficiency, feedback, procedural consistency, and predictability of managerial processes (Deng et al., 2025). Different algorithmic management functions and implementation features may also have different psychological implications (Chen et al., 2026). For example, scheduling, task allocation, and feedback may facilitate coordination and role clarity when implemented with understandable rules and appropriate human oversight. In contrast, intensive monitoring and opaque performance evaluations may heighten perceptions of surveillance and uncertainty. More broadly, practices involving rigid automated decisions, frequent adaptation requirements, restricted employee discretion, and limited opportunities for human explanation may create substantial job demand (Liu et al., 2025). Recent systematic and empirical evidence likewise suggests that the consequences of algorithmic management vary according to the specific configuration of algorithmic control and the organizational context in which it is implemented. Accordingly, this study does not assume that algorithmic management is inherently harmful but focuses on situations in which its demanding and controlling features become particularly salient in the workplace.

Technostress theory provides the primary theoretical framework for this study because its stressor–strain–outcome logic closely maps onto the proposed relationships among algorithmic management, AI anxiety, and work withdrawal (Kotek and Vranjes, 2025; Ragu-Nathan et al., 2008). Within this framework, the demanding features of algorithmic management are conceptualized as technology-related stressors, AI anxiety as psychological strain, and work withdrawal as a potential behavioral response. In contrast, perceived algorithmic transparency is treated as a potential informational resource and boundary condition that may reduce uncertainty by improving employees’ access to and understanding of algorithmic procedures.

Related perspectives provide complementary explanations of how employees may appraise algorithmic management practices. The Job Demands–resources model highlights the balance between demands and resources (Demerouti et al., 2001), conservation of resources theory emphasizes threats to valued resources (Hobfoll, 1989), job insecurity theory emphasizes threats to occupational security, and organizational justice focuses on perceptions of fairness. These perspectives help explain why particular practices may become stressful, whereas technostress theory provides an overarching process linking technology-related demands to psychological strain and behavioral responses.

Therefore, this study extends work-withdrawal research to algorithmic management settings, connects this context with the stressor–strain–outcome logic of technostress theory, and examines perceived algorithmic transparency as a boundary condition for employees’ responses to algorithmic management.

## 2 Literature review and hypothesis development

### 2.1 Algorithmic management and employees’ work withdrawal behavior

With the continuous integration of digital technologies, artificial intelligence, and automated decision-making systems into organizational management processes, firms’ approaches to managing employee behavior are gradually shifting from traditional interpersonal supervision to more data-driven and algorithmic forms of management. Algorithmic systems have increasingly been incorporated into managerial activities and have reshaped how employees’ work is organized, monitored, and evaluated (Kellogg et al., 2020; Lee et al., 2015; Parent-Rocheleau and Parker, 2022; Rosenblat and Stark, 2015). Compared with traditional management approaches, algorithmic management is characterized by greater datafication, automation, and continuity (Jarrahi et al., 2021; Wood et al., 2019).

Algorithmic management refers to the use of algorithmic systems to perform or support managerial functions such as task allocation, work scheduling, behavioral monitoring, performance evaluation, and feedback (Parent-Rocheleau et al., 2024). Although algorithmic management is increasingly associated with AI-enabled technologies, algorithmic systems do not necessarily involve artificial intelligence in a strict sense, as they may also operate through predefined rules or statistical models. Related practices, such as automated decision-making and electronic monitoring, may form part of algorithmic management but are not synonymous with it.

Importantly, algorithmic management is not a homogeneous practice. Different managerial functions and implementation approaches may have different psychological consequences for employees. When algorithmic systems support coordination, provide understandable information, and preserve meaningful human judgment, they may function as enabling resources. In contrast, when they involve intensive monitoring, opaque or rigid decision rules, restricted employee discretion, or limited opportunities for explanation and correction, they may be experienced as greater job demands (Kellogg et al., 2020). Accordingly, employees’ responses to algorithmic management are likely to depend on both the managerial function performed and the manner in which the system is designed and implemented in the workplace.

Work withdrawal behavior refers to a set of negative behavioral responses in which employees voluntarily reduce their connection with work when they experience job stress, dissatisfaction, or threats to their job security. It typically manifests as psychological disengagement, reduced work engagement, decreased proactive behavior, avoidance of work tasks, lateness, absenteeism, and turnover intention (Hanisch and Hulin, 1990; Lehman and Simpson, 1992). As a typical form of counterproductive work behavior, work withdrawal not only affects individual performance and career development, but also undermines team efficiency and increases organizational management costs (Berry et al., 2012; Hom and Kinicki, 2001).

Existing research has shown that when employees develop negative evaluations of their work environment or experience high levels of stress, control, or injustice, they are more likely to reduce their work engagement and adopt withdrawal behaviors as coping responses (Harrison et al., 2006; Podsakoff et al., 2007; Spector et al., 2006).

Drawing on technostress theory, algorithmic management may be associated with work withdrawal when its demand-generating features are prominent. Unlike conventional interpersonal supervision, algorithmic control may involve continuous data collection, automated evaluation, opaque or difficult-to-challenge decision rules, and limited opportunities for human explanation or intervention. These features may increase uncertainty, reduce perceived control, and create technology-related demands, which may, in turn, be associated with psychological and behavioral distancing from the workplace. Accordingly, while recognizing that the consequences of algorithmic management may vary across functions and implementation contexts, greater algorithmic management is expected to be positively associated with employees’ work withdrawal behavior.

*H1*: Algorithmic management is positively associated with employees’ work withdrawal behavior.

### 2.2 The mediating role of AI anxiety

Algorithmic management may be associated with employees’ psychological states and behavioral responses. In traditional management contexts, employees may obtain information about task arrangements and performance evaluations through supervisors, organizational rules, and prior experience. However, under algorithmic management, task allocation, monitoring, feedback, and evaluation may increasingly be mediated by algorithmic systems. When the underlying decision rules are difficult to understand, evaluation outcomes are unpredictable, or opportunities for human explanation are limited, employees may experience greater uncertainty about how managerial decisions are made and how they may affect their future work (Curchod et al., 2020; Rahman, 2021).

AI anxiety refers to feelings of tension, worry, or unease associated with artificial intelligence, particularly concerns about job replacement and difficulties in learning or understanding AI (Li and Huang, 2020). In the present study, AI anxiety represents broader workplace concerns about AI rather than anxiety toward a specific algorithmic management system. This distinction reflects the measurement of the two constructs: algorithmic management refers to management practices used in respondents’ organizations, whereas AI anxiety captures more general concerns about AI-related replacement and learning demands of employees.

Nevertheless, algorithmic management may be associated with the salience of such concerns in the workplace. As task allocation, monitoring, feedback, and performance evaluation become increasingly mediated by algorithmic systems, employees may become more aware of technological changes in managerial control and the need to adapt to technology-enabled work. Opaque decision rules, automated evaluation, and limited opportunities for human explanation may also be associated with greater uncertainty about performance requirements and future work, particularly when employees have difficulty understanding or challenging algorithmic decisions (Curchod et al., 2020; Rahman, 2021). More broadly, organizational research suggests that employees’ reactions to AI are related to the context in which AI-related technologies are introduced and supported (Soomro et al., 2026). Related research on organizational AI adoption further indicates that AI anxiety operates alongside factors such as trust in AI, facilitating conditions, performance expectancy, and perceived response costs in shaping organizational adoption decisions (Maldonado-Canca et al., 2024).

Employees exposed to greater algorithmic involvement in managerial processes may therefore report stronger concerns about technological replacement and their ability to learn and adapt to AI-related technologies. Such an association does not require algorithmic management and AI anxiety to have identical referents; rather, organizational algorithmic practices may constitute a workplace context in which broader AI-related concerns become more salient. Accordingly, greater algorithmic management is expected to be positively associated with anxiety levels.

*H2*: Algorithmic management is positively associated with AI anxiety.

Such AI-related concerns, including concerns about technological replacement, changing skill requirements, and difficulties in understanding or adapting to AI-related technologies, may be associated with greater cognitive and emotional strain and lower work engagement among employees. Related research on job insecurity has shown that job insecurity is associated with poorer job attitudes, organizational attitudes, and mental health, as well as lower commitment and higher turnover intention (Cheng and Chan, 2008; Sverke et al., 2002; Witte, 1999).

In algorithmic management contexts, higher AI anxiety may be associated with more threatening evaluations of the work environment and greater behavioral or psychological withdrawal, including reduced effort, task avoidance, lower proactivity, and psychological disengagement. Similar findings in technology-use research show that stress and exhaustion are associated with reduced usage or avoidance behaviors (Maier et al., 2015). Withdrawal and counterproductive behavior studies also suggest that employees respond to negative work conditions by reducing engagement, increasing absenteeism, or lowering their efforts (Fox et al., 2001; Podsakoff et al., 2007).

*H3*: AI anxiety is positively associated with employees’ work withdrawal behavior.

Based on the stressor–strain–outcome logic of Technostress Theory, the proposed model links the demanding characteristics of algorithmic management with psychological strain and behavioral withdrawal. Specifically, algorithmic management experiences characterized by monitoring, uncertainty, adaptation demands, or opaque evaluation may be associated with higher AI anxiety, which in turn may be associated with greater work withdrawal. Accordingly, algorithmic management is expected to have a positive indirect association with work withdrawal behavior through AI anxiety.

*H4*: Algorithmic management has a positive indirect association with employees’ work withdrawal behavior through AI anxiety.

### 2.3 The moderating role of perceived algorithmic transparency

Perceived algorithmic transparency refers to employees’ subjective perceptions of the extent to which information about the organizational algorithmic system used to manage their work is available to them and how well they understand the system’s operation. Therefore, the construct is perceived by employees rather than being an objective technical property of the system itself. Perceived algorithmic transparency may include two closely related elements: First, information availability, which refers to whether employees believe that they can access information about the system, its operating procedures, and the criteria used in algorithm-supported management. Second, comprehensibility refers to whether employees believe that the available information enables them to understand how the system affects task allocation, monitoring, performance evaluation and feedback. This construct should be distinguished from other related concepts such as self-efficacy. Technical explainability concerns whether the operation or output of an algorithm can be explained in an interpretable way. Procedural transparency concerns the disclosure of the procedures and criteria through which decisions are made. Contestability refers to employees’ ability to question or challenge algorithm-supported decisions, and human oversight concerns the extent to which human decision-makers review or override system outputs. The present measure primarily captures perceived information availability and comprehensibility. It does not directly measure objective disclosure, technical explainability, procedural fairness, contestability, or human oversight.

Within the present framework, perceived algorithmic transparency is not conceptualized as technostress. Rather, it is treated as a potential informational resource and boundary condition associated with employees’ access to and understanding of information about algorithmic procedures and evaluation criteria, and potentially with lower perceived uncertainty and greater perceived control. When employees can access and understand information about algorithmic procedures, their perceptions of the system may change accordingly (Durcikova and Gray, 2009; Li et al., 2025). In algorithmic management contexts, higher perceived transparency may be associated with a greater understanding of how algorithmic systems relate to task allocation, performance evaluation, and feedback. Prior research suggests that transparency and explainability are associated with users’ trust, comprehension, and acceptance of algorithmic or AI-based systems (Kizilcec, 2016; Shin, 2021).

When perceived algorithmic transparency is low, employees may have greater difficulty understanding how algorithmic decisions are made and may report greater uncertainty and perceived lack of control (Curchod et al., 2020; Rahman, 2021). In contrast, higher perceived transparency may provide employees with greater access to understandable information about system procedures and evaluation criteria, thereby reducing uncertainty and perceived uncontrollability of AI-generated performance evaluations. However, greater transparency may not always reduce anxiety. By making algorithmic processes more visible, employees’ awareness of intensive monitoring, automated evaluation, or potential job substitution may increase, thereby making technology-related threats more salient. Thus, perceived algorithmic transparency may have competing implications for reducing anxiety. In the present study, we focus on its potential uncertainty-reducing role and therefore expect a positive association between algorithmic management and AI anxiety to be weaker at higher levels of perceived algorithmic transparency.

*H5*: Perceived algorithmic transparency negatively moderates the positive association between algorithmic management and AI anxiety, such that the association weakens as perceived algorithmic transparency increases.

Because perceived algorithmic transparency is expected to weaken the association between algorithmic management and AI anxiety, the indirect association between algorithmic management and work withdrawal behavior through AI anxiety should vary across different levels of perceived algorithmic transparency. Specifically, when perceived algorithmic transparency is higher, the association between algorithmic management and AI anxiety is expected to be weaker, which, in turn, should correspond to a weaker indirect association with work withdrawal behavior through AI anxiety.

*H6*: The indirect association between algorithmic management and employee work withdrawal behavior through AI anxiety is weaker when perceived algorithmic transparency is higher. The theoretical model used in this study is shown in Figure 1.

**Figure 1:**
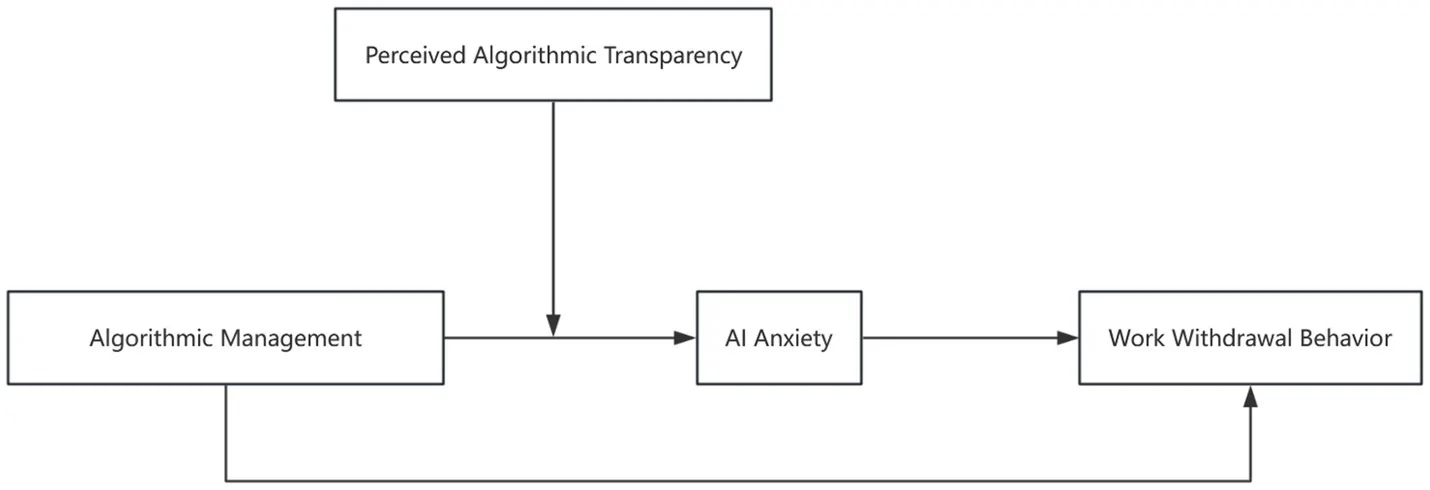
Proposed theoretical model.

## 3 Materials and methods

### 3.1 Research sample and data collection

Data were collected from February to April 2026 from employees of Internet sector enterprises in several provinces in China, including Zhejiang, Hubei, and Fujian. Participants were recruited through two channels: internal employee groups and organizational contacts, and online survey platforms. Of the 435 individuals who responded to the eligibility screening question, 260 were recruited through the first channel and 175 through the second. Respondents who completed the full questionnaire received RMB 2 as a participation incentive. Before entering the main questionnaire, participants were asked: “Does your organization use algorithmic management to supervise your daily work?” Only respondents who answered “Yes” were allowed to continue. Of the 435 individuals who responded to the screening question, 55 were excluded at this stage, leaving 380 eligible respondents. The questionnaire included two response-quality checks: an instructional attention-check item requiring respondents to select a specified response option and a factual-check item concerning the capital city of Hubei Province, China. After excluding 34 respondents who failed these quality checks, the final analytical sample consisted of 346 employees. A sensitivity power analysis for the interaction test indicated that, with *N* = 346 and α = 0.05, the study had 80% power to detect a local effect of approximately f^2^ = 0.023 and 90% power to detect an approximate f^2^ = 0.031. Thus, 87.36% of the individuals who responded to the screening question met the eligibility criteria, and 91.05% of the eligible respondents were retained in the final analytical sample. To reduce duplicate participation, the survey platform restricted each device and WeChat account to one submission, and respondents reported their frequency of personal AI use in work or daily activities. AI-use frequency was treated as a sample characteristic and control variable, rather than an eligibility criterion. The personal use of AI tools is conceptually distinct from exposure to algorithmic management because employees may be monitored, scheduled, evaluated, or assigned tasks through algorithmic systems without actively operating AI tools. Accordingly, a low frequency of personal AI use was not considered evidence of insufficient exposure to algorithmic management.

Among the final analytical sample, 47.1% of the respondents were male and 52.9% were female. Most respondents were between 26 and 35 years of age. Regarding educational background, 49.7% of the respondents held a bachelor’s degree. Regarding job levels, the sample primarily comprised frontline employees and supervisors. The detailed sample characteristics are shown in Table 1.

**Table 1:** Sample characteristics.

Variable	Category	Frequency (n)	Proportion
Gender	Male	163	47.10%
	Female	183	52.90%
Age	25 or below	51	14.70%
	26–30	101	29.20%
	31–35	88	25.40%
	36–40	49	14.20%
	41–45	35	10.10%
	46 or above	22	6.40%
Education	High school or below	42	12.10%
	Junior college	89	25.70%
	Bachelor’s degree	172	49.70%
	Graduate or above	43	12.40%
Tenure	Less than 1 year	37	10.70%
	1–3 years	101	29.20%
	4–6 years	86	24.90%
	7–10 years	66	19.10%
	More than 10 years	56	16.20%
Job level	Employee	219	63.30%
	Front-line supervisor	75	21.70%
	Middle manager	42	12.10%
	Senior manager	10	2.90%
AI-use frequency	Never	39	11.30%
	Rarely	60	17.30%
	Sometimes	130	37.60%
	Frequently	85	24.60%
	Highly frequent	32	9.20%

Gender and age were included to account for demographic differences, education and AI-use frequency to account for potential differences in familiarity with and adaptation to digital and AI technologies, and organizational tenure and job level to account for differences in organizational experience and role context. These variables were included as covariates because such background characteristics may be associated with employees’ technology-related perceptions and work responses.

### 3.2 Variable measurement

The questionnaire was administered in Chinese. The measures were based on established scales and prior Chinese-language versions or applications reported in the literature. Algorithmic management was assessed using a 7-point agreement scale ranging from 1 (strongly disagree) to 7 (strongly agree). AI anxiety and perceived algorithmic transparency were assessed using 5-point agreement scales ranging from 1 (strongly disagree) to 5 (strongly agree), and work withdrawal behavior was assessed using a 5-point frequency scale ranging from 1 (never) to 5 (frequently). The original response formats of the established scales were retained to maintain consistency with the published versions. Following the demographic, screening, and attention-check items, the focal measures were presented in a fixed order: algorithmic management, AI anxiety, perceived algorithmic transparency, and work withdrawal behavior. The order of the focal measures was not counterbalanced.

Algorithmic management was measured using the 12-item scale adapted and validated by Liu et al. (2025) based on the algorithmic management measure originally developed by Parent-Rocheleau et al. (2024). The scale covers four managerial functions: monitoring, task allocation, work scheduling, and performance and compensation, with three items for each function. Respondents rated the items on a 7-point agreement scale ranging from 1 (strongly disagree) to 7 (strongly agree). Representative items include “The company uses an algorithmic system to identify whether my work process complies with operational standards and provides improvement suggestions” and “The company uses an algorithmic system to schedule my weekly/monthly work tasks.” Cronbach’s alpha for the 12-item scale was 0.902 and McDonald’s omega was 0.903.

AI anxiety was measured using six items from Li and Huang (2020), covering job-replacement anxiety and learning anxiety. The learning-anxiety items focus on perceived difficulty in understanding, learning, or mastering AI-related knowledge and may therefore also contain an element of perceived AI-related competence. Participants responded on a 5-point agreement scale ranging from 1 (strongly disagree) to 5 (strongly agree) (Wang and Liu, 2025). Representative items were “I am worried that artificial intelligence will replace my job in the future” and “Understanding AI algorithms requires a high level of talent and skills, which is very difficult for me.” The items referred to artificial intelligence and AI-related work demands in general rather than a specifically named algorithmic management system in the respondent’s organization. Cronbach’s alpha for the 6-item measure was 0.848 and McDonald’s omega was 0.849.

Work withdrawal behavior was measured using the 12-item scale originally developed by Lehman and Simpson (1992). The Chinese item wording used in the present survey was adapted with reference to Zhao and Jiao (2023). The scale includes eight items reflecting psychological withdrawal and four items reflecting physical withdrawal from work. Respondents reported how frequently they engaged in each behavior during the previous month using a 5-point frequency scale ranging from 1 (never) to 5 (Frequently). Representative items were “I have had thoughts of being absent from work” and “I have left my workplace without permission.” Cronbach’s alpha for the 12-item measure was 0.892 and McDonald’s omega was 0.892.

Perceived algorithmic transparency was measured using three items based on Durcikova and Gray (2009), with the Chinese wording and adaptation to the algorithmic-work context informed by Gao et al. (2025). Participants responded on a 5-point agreement scale ranging from 1 (strongly disagree) to 5 (strongly agree). Representative items were “I clearly understand how the algorithmic system used by the company to manage my work operates” and “I can access information about the algorithmic system used by the company to manage my work.” These items capture employees’ perceived understanding of and access to information about the algorithmic system. Cronbach’s alpha for the three-item measure was 0.749. McDonald’s omega was 0.752.

For subsequent analyses, the score for each focal variable was calculated as the mean of the relevant items.

## 4 Results

### 4.1 Confirmatory factor analysis

To examine the discriminant validity among the variables, this study conducted a confirmatory factor analysis (CFA) using AMOS software on the four latent constructs: algorithmic management, AI anxiety, work withdrawal behavior, and perceived algorithmic transparency. Furthermore, the model fit of the four-factor model was compared with that of the three-, two-, and single-factor models.

As shown in Table 2, the four-factor model demonstrated the best fit: χ^2^/df = 1.501, GFI = 0.890, CFI = 0.949, TLI = 0.945, RMSEA = 0.038, indicating a good overall model fit. In comparison, the fit indices of the three-, two-, and single-factor models decreased to varying degrees, with the single-factor model showing the poorest fit. These results support the empirical distinctiveness of algorithmic management, AI anxiety, work withdrawal behavior, and perceived algorithmic transparency, although relatively strong associations among some constructs were observed. Given the multidimensional content of several measures, additional CFA comparisons were conducted to examine whether the corresponding subdimensions needed to be modeled separately. For algorithmic management, the correlated four-factor model did not provide a significant improvement over the one-factor model, Δχ^2^(6) = 9.705, *p* = 0.138, and the one-factor model showed a slightly lower AIC (100.290 vs. 102.585). Similarly, for AI anxiety, the correlated two-factor model did not improve fit relative to the one-factor model, Δχ^2^(1) = 0.551, *p* = 0.458, and the one-factor model again yielded a slightly lower AIC (42.822 vs. 44.271). For work withdrawal behavior, the correlated two-factor model provided a modest improvement over the one-factor model, Δχ^2^(1) = 5.238, *p* = 0.022. However, the psychological and physical withdrawal factors were highly correlated (r = 0.947), while the one-factor model also showed a very good absolute fit (χ^2^/df = 1.146, CFI = 0.994, TLI = 0.993, RMSEA = 0.021). Overall, these results did not suggest that treating the subdimensions as separate variables was necessary for the focal analyses. Accordingly, the overall item mean scores were retained, while the multidimensional content of the measures was acknowledged.

**Table 2:** Confirmatory factor analysis results.

Model	X^2^	df	X^2^/df	GFI	CFI	TLI	RMSEA
Four-factor model	734.047	489	1.501	0.890	0.949	0.945	0.038
Three-factor model	884.304	492	1.797	0.856	0.918	0.912	0.048
Two-factor model	1128.406	494	2.284	0.792	0.867	0.858	0.061
Single-factor model	1377.387	495	2.783	0.767	0.823	0.812	0.070

Four-factor model: algorithmic management, AI anxiety, work withdrawal behavior, and perceived algorithmic transparency; three-factor model: algorithmic management + AI anxiety, work withdrawal behavior, and perceived algorithmic transparency; two-factor model: algorithmic management + AI anxiety + work withdrawal behavior, and perceived algorithmic transparency; single-factor model: algorithmic management + AI anxiety + work withdrawal behavior + perceived algorithmic transparency.

As shown in Table 3, all standardized factor loadings exceeded 0.50, and the composite reliability values for the four constructs were greater than 0.70, indicating good reliability. The AVE values ranged from 0.409 to 0.501, with several constructs falling below the conventional 0.50 benchmark. Overall, the measurement results support acceptable reliability, although the convergent validity of some constructs should be interpreted with caution.

**Table 3:** Measurement reliability and convergent validity.

Construct	Item	Standardized loading	CR	AVE	√AVE
AM	AM1	0.596	0.902	0.437	0.661
	AM2	0.666			
	AM3	0.664			
	AM4	0.722			
	AM5	0.643			
	AM6	0.722			
	AM7	0.62			
	AM8	0.648			
	AM9	0.594			
	AM10	0.654			
	AM11	0.631			
	AM12	0.748			
AIA	AIA1	0.674	0.849	0.484	0.696
	AIA2	0.718			
	AIA3	0.627			
	AIA4	0.74			
	AIA5	0.672			
	AIA6	0.738			
WWB	WWB1	0.621	0.892	0.409	0.639
	WWB2	0.662			
	WWB3	0.579			
	WWB4	0.656			
	WWB5	0.666			
	WWB6	0.628			
	WWB7	0.684			
	WWB8	0.679			
	WWB9	0.611			
	WWB10	0.646			
	WWB11	0.633			
	WWB12	0.596			
PAT	PAT1	0.751	0.750	0.501	0.708
	PAT2	0.714			
	PAT3	0.656			

AM, algorithmic management; AIA, AI anxiety; WWB, work withdrawal behavior; PAT, perceived algorithmic transparency.

Beyond convergent validity, discriminant validity was assessed using the Fornell–Larcker criterion, heterotrait–monotrait ratio (HTMT), and comparisons among alternative CFA models. The latent factor correlations and HTMT bootstrap confidence intervals are reported in Table 4. The results provided mixed evidence regarding discriminant validity. The Fornell–Larcker criterion was not met for three construct pairs: algorithmic management–AI anxiety, algorithmic management–work withdrawal behavior, and AI anxiety–work withdrawal behavior. In contrast, all HTMT point estimates were below the conservative 0.85 threshold. However, the 95% bootstrap confidence intervals for algorithmic management–AI anxiety and AI anxiety–work withdrawal behavior extended slightly above 0.85, indicating some overlap between these constructs. In addition, the hypothesized four-factor model showed a better overall fit than the alternative models. Notably, the three-factor model also showed an acceptable fit (CFI = 0.918, RMSEA = 0.048). Taken together, these results provide qualified support for the empirical distinction among the focal constructs while also indicating some degree of overlap among algorithmic management, AI anxiety, and work withdrawal behavior (Table 2).

**Table 4:** Heterotrait–Monotrait ratios among the study constructs.

Construct pair	Latent-factor correlation	HTMT	95% bootstrap CI
AM–AIA	0.832	0.822	[0.770, 0.867]
AM–WWB	0.748	0.736	[0.673, 0.794]
AM–PAT	−0.271	0.273	[0.176, 0.397]
AIA–WWB	0.846	0.838	[0.787, 0.883]
AIA–PAT	−0.377	0.374	[0.257, 0.485]
WWB–PAT	−0.303	0.307	[0.198, 0.423]

AM, algorithmic management; AIA, AI anxiety; WWB, work withdrawal behavior; PAT, perceived algorithmic transparency. Latent-factor correlations were obtained from the four-factor CFA model. HTMT confidence intervals were based on 5,000 bootstrap samples.

### 4.2 Common method bias test

Because all focal variables were collected from the same respondents at a single point in time, common source variance cannot be fully ruled out. To reduce potential response bias, respondents were informed that their responses would be anonymous and confidential, that the data would be used solely for academic research, and that there were no correct or incorrect answers to the questions. Different response formats were used for the focal measures (Table 5).

**Table 5:** Model fit after adding the common method factor.

Model	X2	df	X2/df	GFI	CFI	TLI	RMSEA
Four-factor model	734.047	489.000	1.501	0.890	0.949	0.945	0.038
Four-factor +cmv	666.574	459.000	1.452	0.899	0.956	0.950	0.036
Difference	67.473	30.000	0.049	0.009	0.007	0.005	0.002

Harman’s single-factor test showed that the first unrotated factor accounted for 36.497% of the total variance. In addition, a latent common method factor was added to the four-factor model. The method factor was specified as orthogonal to the four substantive factors, with one method factor loading fixed to 1.0 for scale identification and the remaining loadings estimated freely. The model converged successfully and showed an acceptable fit (χ^2^/df = 1.452, GFI = 0.899, CFI = 0.956, TLI = 0.950, RMSEA = 0.036). These analyses did not indicate that a single factor accounted for the majority of the covariance. Nevertheless, common-source variance cannot be excluded and should be considered when interpreting these findings.

### 4.3 Descriptive statistics and correlation analysis

This study conducted a correlation analysis of the focal variables, including algorithmic management (AM), AI anxiety (AIA), work withdrawal behavior (WWB), and perceived algorithmic transparency (PAT) (Table 6). AM was positively correlated with AIA (r = 0.721, *p* < 0.01) and WWB (r = 0.659, *p* < 0.01), and AIA was positively correlated with WWB (r = 0.729, *p* < 0.01). Additional analyses showed that AM was similarly correlated with replacement anxiety (r = 0.675, *p* < 0.001) and learning anxiety (r = 0.673, *p* < 0.001). PAT was negatively correlated with AM (r = −0.222, *p* < 0.01), AIA (r = −0.295, *p* < 0.01), and WWB (r = −0.254, *p* < 0.01).

**Table 6:** Descriptive statistics and correlation matrix.

Variable	M	SD	1	2	3	4	5	6	7	8	9	10
1 Gender	1.53	0.5	1									
2 Age	2.95	1.415	0.105	1								
3 Education	2.62	0.853	0.06	−0.016	1							
4 Tenure	3.01	1.25	−0.04	−0.01	−0.09	1						
5 Job level	1.55	0.816	0.029	−0.003	0.112*	−0.022	1					
6 AI usage frequency	3.03	1.115	0.011	0.007	0.013	−0.058	0.025	1				
7 AM	4.05	0.75	−0.066	−0.043	−0.087	0.025	−0.049	0.075	1			
8 AIA	2.85	0.53	0.03	−0.02	−0.004	−0.04	−0.05	0.035	0.721**	1		
9 WWB	2.98	0.49	0.032	−0.037	−0.08	−0.064	−0.072	0.056	0.659**	0.729**	1	
10 PAT	3.11	0.6	−0.03	−0.112*	−0.037	0.052	0.051	0.025	−0.222**	−0.295**	−0.254**	1

*N* = 346. **p < 0.01. *p < 0.05. AM, algorithmic management; AIA, AI anxiety; WWB, work withdrawal behavior; PAT, perceived algorithmic transparency.

To further examine multicollinearity, the variance inflation factor (VIF) and tolerance values were tested for all predictor variables. The results showed that the VIF values ranged from 1.003 to 2.082 (well below the threshold of 5), and the tolerance values ranged from 0.480 to 0.951 (well above 0.1), indicating that no serious multicollinearity problem existed in the data.

### 4.4 Hypothesis testing

Before conducting the regression analyses, the data were examined for distributional characteristics, outliers, and regression assumptions. The skewness of the focal variables ranged from −0.066 to 0.163, and the kurtosis ranged from −0.392 to 0.206, showing relatively limited departures from normality. Outlier screening identified one potentially unusual AI anxiety score (|z| = 3.42). The Mahalanobis distance was used to screen for multivariate outliers, and no cases exceeded the χ^2^ critical value at *p* < 0.001. However, the maximum Cook’s distance was low (0.0449), suggesting that no single observation exerted undue influence on the regression estimates. For the focal regression equation, the standardized residuals ranged from −3.218 to 3.044, and the residual plot showed no clear curvilinear or funnel-shaped pattern, suggesting that linearity and homoscedasticity were generally acceptable. The Durbin–Watson statistic was 1.883, suggesting no substantial first-order residual autocorrelation in the model.

#### 4.4.1 Direct and indirect association analysis

Hierarchical regression analysis was conducted to examine the hypothesized associations (Table 7). After adjusting for the control variables, algorithmic management was positively associated with employees’ work withdrawal behavior (β = 0.661, *p* < 0.001), thus supporting H1. Algorithmic management was also positively associated with AI anxiety (β = 0.733, *p* < 0.001), thus supporting H2. When algorithmic management and AI anxiety were simultaneously included in the model, AI anxiety remained positively associated with work withdrawal behavior (β = 0.523, *p* < 0.001), whereas the association between algorithmic management and work withdrawal behavior remained statistically significant but decreased (β = 0.278, *p* < 0.001). Thus, H3 is supported. Table 7 reports the unstandardized coefficients (B) with standard errors, whereas the standardized coefficients (β) are reported in the text.

**Table 7:** Results of the hierarchical regression test.

Variables	AIA				WWB		
	Model 1	Model 2	Model 3	Model 4	Model 5	Model 6	Model 7
1 Gender	0.034 (0.058)	0.078 (0.040)	0.073 (0.039)	0.064 (0.038)	0.039 (0.053)	0.075 (0.040)	0.038 (0.035)
2 Age	−0.009 (0.020)	0.002 (0.014)	−0.004 (0.014)	−0.002 (0.014)	−0.015 (0.019)	−0.006 (0.014)	−0.007 (0.012)
3 Education	−0.003 (0.034)	0.033 (0.023)	0.028 (0.023)	0.029 (0.023)	−0.047 (0.031)	−0.017 (0.024)	−0.033 (0.021)
4 Tenure	−0.020 (0.023)	−0.026 (0.016)	−0.023 (0.016)	−0.022 (0.015)	−0.027 (0.021)	−0.032 (0.016)*	−0.020 (0.014)
5 Job level	−0.034 (0.035)	−0.015 (0.024)	−0.011 (0.024)	−0.001 (0.024)	−0.040 (0.032)	−0.024 (0.024)	−0.017 (0.021)
6 AI usage frequency	0.016 (0.026)	−0.012 (0.018)	−0.009 (0.018)	−0.007 (0.017)	0.024 (0.024)	0.001 (0.018)	0.007 (0.016)
7 AM.	—	0.519 (0.027)***	0.497 (0.027)***	0.498 (0.026)***	—	0.433 (0.027)***	0.182 (0.034)***
8 AIA	—	—	—	—	—	—	0.483 (0.048)***
9 PAT	—	—	−0.119 (0.034)***	−0.112 (0.033)***	—	—	—
10 AM*PAT	—	—	—	−0.174 (0.041)***	—	—	—
ΔF	0.431	380.688***	12.264***	17.713***	1.261	262.371***	101.595***
R^2^	0.008	0.533	0.550	0.572	0.022	0.449	0.577
ΔR^2^	0.008	0.526	0.016	0.023	0.022	0.427	0.128

The entries are unstandardized regression coefficients (B), with standard errors in parentheses. AM, algorithmic management; AIA, AI anxiety; WWB, work withdrawal behavior; PAT, perceived algorithmic transparency. *p < 0.05, **p < 0.01, ***p < 0.001.

To further examine the hypothesized indirect association, PROCESS v4.1 Model 4 with 5,000 bootstrap samples was used. The primary PROCESS model was estimated without covariates using conventional ordinary least squares (OLS) standard error. The estimated indirect association between algorithmic management and work withdrawal behavior through AI anxiety was 0.2495 (BootSE = 0.0269, 95% bootstrap CI [0.1985, 0.3044]). As the bootstrap confidence interval did not include zero, the indirect association was significant. The association between algorithmic management and work withdrawal behavior remained statistically significant after AI anxiety was included in the model (B = 0.1820, SE = 0.0335, t = 5.4338, *p* < 0.001, 95% CI: [0.1161, 0.2479]). This pattern is conventionally described as partial statistical mediation. However, given its cross-sectional design, it should not be interpreted as evidence of separate direct and indirect causal pathways between these variables. The remaining associations may reflect omitted variables, measurement overlap, reverse or reciprocal relationships, and common-source covariance. Thus, H4 was supported at the level of statistical significance (Table 8).

**Table 8:** Bootstrap results of mediation effect.

Pathway	Effect	SE/BootSE	95% CI Lower	95% CI Upper
Total effect	0.4316	0.0265	0.3793	0.4838
Direct effect	0.1820	0.0335	0.1161	0.2479
Total indirect effect	0.2495	0.0269	0.1985	0.3044
AM → AIA → WWB	0.2495	0.0269	0.1985	0.3044

The effects reported in this table are unstandardized PROCESS estimates. SE is reported for the total and direct effects, whereas BootSE is reported for the indirect effects. Bootstrap samples = 5,000. AM, algorithmic management; AIA, AI anxiety; WWB, work withdrawal behavior.

#### 4.4.2 Moderating effect analysis

To examine whether perceived algorithmic transparency moderated the association between algorithmic management and AI anxiety, an interaction term between algorithmic management and perceived algorithmic transparency was constructed after mean centering the variables. The interaction term was significantly and negatively associated with AI anxiety (β = −0.152, *p* < 0.001), indicating that the positive association between algorithmic management and AI anxiety was weaker at higher levels of perceived algorithmic transparency. Thus, H5 is supported. Simple slope analyses further showed that this positive association was significant at both low and high levels of perceived algorithmic transparency but was stronger when perceived algorithmic transparency was low. The corresponding estimates are reported in Table 9 and the interaction pattern is illustrated in Figure 2.

**Table 9:** Simple-slope analysis of the moderating effect.

PAT level	B	SE	t	p	95% CI
Low (−1 SD)	0.5942	0.0357	16.6438	<0.001	[0.5240, 0.6645]
High (+1 SD)	0.3836	0.0356	10.7741	<0.001	[0.3136, 0.4536]

B, unstandardized coefficient; SE, standard error; PAT, perceived algorithmic transparency; CI, confidence interval.

**Figure 2:**
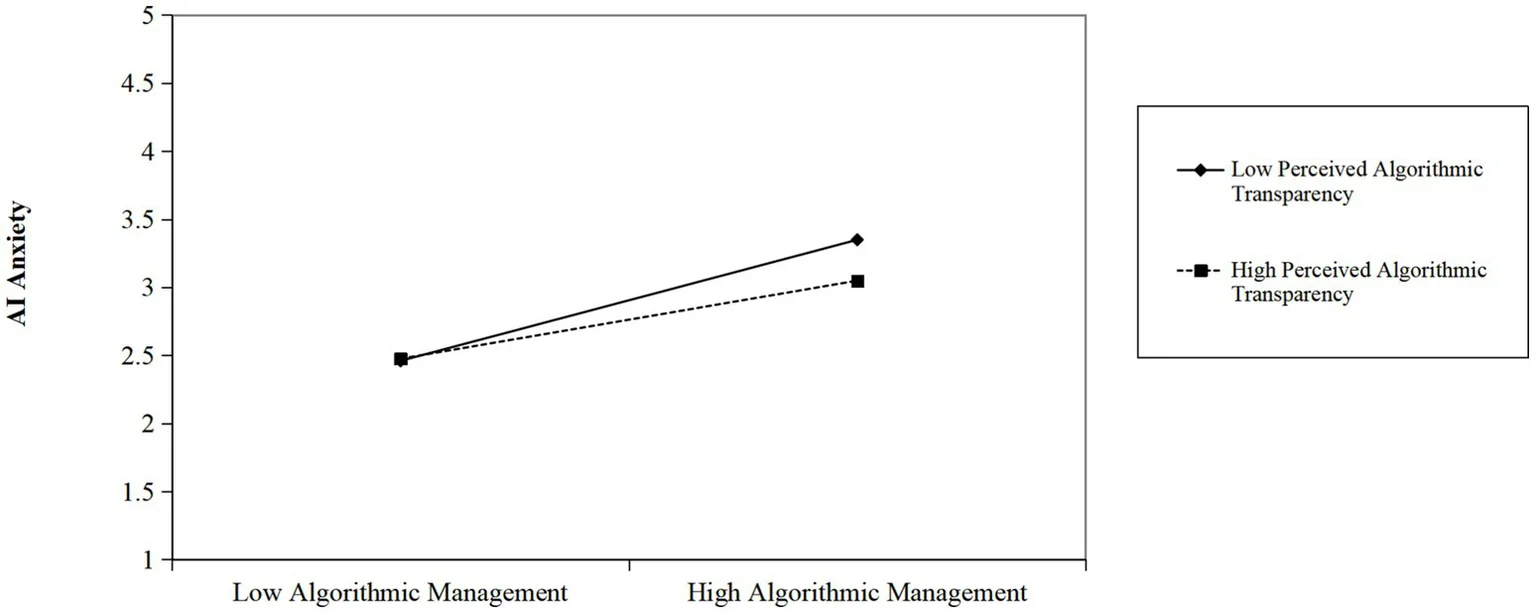
Moderating pattern of perceived algorithmic transparency.

#### 4.4.3 Robustness check

To assess whether the results were driven by respondents with no or limited personal AI use, we repeated the core regression and indirect association analyses after excluding participants who reported never or rarely using AI, resulting in a subsample of 247 respondents. The results remained substantively unchanged. Algorithmic management remained positively associated with AI anxiety and work-withdrawal behavior. Moreover, the estimated indirect association between algorithmic management and work withdrawal behavior through AI anxiety remained statistically significant (indirect association = 0.2517, BootSE = 0.0341, 95% CI [0.1880, 0.3226]). The association between algorithmic management and work withdrawal behavior remained statistically significant after AI anxiety was included in the model (B = 0.2183, *p* < 0.001). These results support the robustness of the core direct and indirect associations after excluding respondents with no or limited personal AI use.

Additional sensitivity analyses were conducted to examine whether the findings depended on the inclusion or coding of control variables. For PROCESS Model 4, the estimated indirect association was 0.2495 without covariates, 0.2508 with control variables, and 0.2522 when ordinal control variables were dummy coded. For PROCESS Model 7, the index of moderated mediation was −0.0864 without covariates, −0.0838 with the six control variables, and −0.0890 with dummy-coded ordinal control. The AM × PAT interaction also remained negative and statistically significant across these specifications, indicating that the substantive conclusions were stable.

#### 4.4.4 Conditional indirect association analysis

To examine the conditional indirect association, PROCESS v4.1 Model 7 was used, with perceived algorithmic transparency specified as a first-stage moderator. The analysis used 5,000 bootstrap samples to construct 95% bootstrap confidence intervals, and the primary model was estimated without covariates using conventional OLS standard errors. The results in Table 10 show that the estimated indirect association between algorithmic management and work withdrawal behavior through AI anxiety varied across levels of perceived algorithmic transparency. When perceived algorithmic transparency was low, the estimated indirect association was 0.2902 (BootSE = 0.0316, 95% bootstrap CI [0.2317, 0.3542]). At the mean level of perceived algorithmic transparency, the estimated indirect association was 0.2388 (BootSE = 0.0245, 95% bootstrap CI [0.1924, 0.2898]). When perceived algorithmic transparency was high, the estimated indirect association decreased to 0.1873 (Boot SE = 0.0233, 95% bootstrap CI [0.1454, 0.2364]). In addition, the index of moderated mediation was −0.0864 (Boot SE = 0.0218, 95% bootstrap CI [−0.1301, −0.0462]), which did not include zero. These results indicate that the estimated indirect association between algorithmic management and work withdrawal behavior through AI anxiety was weaker at higher levels of perceived algorithmic transparency. Thus, H6 was supported at the level of conditional indirect association.

**Table 10:** Conditional indirect effects and index of moderated mediation.

Level of perceived algorithmic transparency	Estimate	BootSE	95% CI LLCI	95% CI ULCI
Low (−1 SD)	0.2902	0.0316	0.2317	0.3542
Mean	0.2388	0.0245	0.1924	0.2898
High (+1 SD)	0.1873	0.0233	0.1454	0.2364
Index of moderated mediation	−0.0864	0.0218	−0.1301	−0.0462

Effects reported in this table are unstandardized PROCESS estimates based on 5,000 bootstrap samples. AM, algorithmic management; AIA, AI anxiety; WWB, work withdrawal behavior; PAT, perceived algorithmic transparency; LLCI, lower limit of the 95% confidence interval; ULCI, upper limit of the 95% confidence interval.

## 5 Discussion

This study examined the relationships among algorithmic management, AI anxiety, perceived algorithmic transparency, and employees’ work withdrawal behavior. The results showed that algorithmic management was positively associated with both AI anxiety and work withdrawal behavior, while AI anxiety was positively associated with work withdrawal. The bootstrap analysis further identified a statistically significant indirect association through AI anxiety, and perceived algorithmic transparency weakened the positive association between algorithmic management and AI anxiety, as well as the estimated indirect association. Because the data were collected from the same respondents at one point in time, these findings should be interpreted as a pattern of associations consistent with the proposed theoretical model rather than as evidence of a demonstrated causal sequence.

The positive association between algorithmic management and AI anxiety may be understood in relation to the distinctive manner in which algorithmic systems organize and exercise managerial control. Compared with many conventional workplace stressors, algorithmic management can embed monitoring, task allocation, scheduling, performance evaluation, and feedback into continuous data collection and automated decision processes. Therefore, employees may experience managerial control as persistent, data-driven, difficult to observe, and difficult to question. In particular, opaque evaluation rules, limited human explanation, and uncertainty about how employee data are translated into performance outcomes may be associated with greater concerns about technological replacement, changing skill requirements, and employees’ future occupational values. This interpretation is consistent with research showing that algorithmic control can create information and power asymmetries and limit employees’ ability to understand or challenge managerial decisions (Curchod et al., 2020; Kellogg et al., 2020; Rahman, 2021). This is also consistent with technostress theory, which suggests that technology-related demands may become psychologically burdensome when employees perceive a limited capacity to understand, predict, or control them (Ragu-Nathan et al., 2008; Salanova et al., 2013). Importantly, this interpretation does not imply that algorithmic management is inherently detrimental, as its consequences may vary according to how particular systems are designed and implemented. At the same time, the relatively strong correlation between algorithmic management and AI anxiety (r = 0.721) warrants caution. Given the cross-sectional, same-source design and some overlap in the measurement evidence, common-source covariance and construct overlap remain plausible alternative explanations for part of this association; accordingly, its magnitude should be interpreted cautiously, rather than as direct evidence of the technostress mechanism.

The positive association between AI anxiety and work withdrawal is consistent with previous research linking job insecurity, technological stress, and unfavorable work conditions to reduced engagement, avoidance, absenteeism, and turnover-related responses (Cheng and Chan, 2008; Harrison et al., 2006; Podsakoff et al., 2007; Sverke et al., 2002). One plausible interpretation is that concerns about job displacement, difficulties in learning or understanding AI, and uncertainty about future occupational value are associated with the depletion of employees’ cognitive and emotional resources. Under such conditions, psychological disengagement, reduced effort, or avoidance may serve as ways of distancing oneself from a work environment perceived as uncertain or threatening. Nevertheless, work withdrawal may also reflect other unmeasured psychological and organizational factors.

The relative size of the standardized coefficients provides additional context for interpreting the findings. Algorithmic management showed a comparatively strong positive association with AI anxiety (β = 0.733), and AI anxiety was positively associated with work withdrawal after algorithmic management was included in the model (β = 0.523). The standardized coefficient linking algorithmic management to work withdrawal decreased from β = 0.661 to β = 0.278 after AI anxiety was included. This attenuation indicates that a substantial portion of the observed association between algorithmic management and work withdrawal was statistically shared with AI anxiety. However, the remaining association should not be interpreted as proof that algorithmic management operates through separate or additional causal mechanisms. This may reflect omitted psychological or organizational variables, overlap between the measures, reciprocal relationships, measurement error, or common-source covariance. Moreover, because these coefficients were estimated using cross-sectional self-reported data, their relative magnitudes should not be interpreted as evidence of temporal or causal importance.

The interaction results suggest that employees’ perceived access to and understanding of information about algorithmic systems may be relevant to the strength of the association between algorithmic management and AI anxiety. When employees perceive algorithmic procedures and evaluation criteria as more understandable, algorithmic management may appear less unpredictable and uncontrollable. However, transparency should not be assumed to have uniformly positive consequences. Greater visibility of algorithmic systems may also make employees more aware of intensive monitoring, automated evaluation practices and potential threats to their occupational roles. Thus, the buffering pattern observed in this study may reflect the uncertainty-reducing role of perceived understandable information rather than indicating that transparency is universally beneficial in reducing uncertainty. This interpretation is consistent with prior research linking access to understandable system information with users’ trust, comprehension, and acceptance of algorithmic systems (Durcikova and Gray, 2009; Kizilcec, 2016; Shin, 2021). Simultaneously, perceived algorithmic transparency is not equivalent to objective technical explainability, procedural fairness, data accuracy, contestability, organizational accountability, or effective human oversight. Transparency may have limited value when the disclosed information is highly technical, incomplete, or cannot be used to question an inaccurate or unfair decision. Accordingly, the findings should not be interpreted as indicating that greater disclosure alone will necessarily reduce AI anxiety or work withdrawal behavior.

### 5.1 Theoretical contributions

First, this study extends research on employees’ work withdrawal behavior by examining a distinctive technology-mediated managerial context. Previous studies primarily explain work withdrawal in relation to job stress, organizational justice, psychological contract breach, burnout, leadership, and organizational support. Algorithmic management introduces additional features because managerial direction, monitoring, evaluation, and feedback may be embedded in continuous data collection, automated procedures, and decision rules that employees cannot easily observe, interpret, or challenge. Therefore, the present study highlights technology-mediated control and information asymmetry as relevant contextual factors associated with employees’ psychological and behavioral disengagement.

Second, this study applies the stressor–strain–outcome logic of technostress theory to algorithmic management without assuming that it is inherently stressful. The theoretical argument distinguishes the broader managerial system from the specific features that may operate as technology-related demands. Continuous monitoring, opaque decision criteria, unpredictable automated evaluation, adaptation requirements, and restricted human influence may be experienced as demands, whereas decision support, timely feedback, and procedural consistency may function as resources. By examining AI anxiety as a psychological strain, this study links demanding experiences of algorithmic management with employees’ work withdrawal behavior within a technostress framework.

Third, this study contributes to a more conditional understanding of algorithmic management by examining perceived algorithmic transparency as a boundary condition. Rather than assuming that employees respond uniformly to algorithmic management, the study shows that the association between algorithmic management and AI anxiety varies with employees’ perceived access to and understanding of information about algorithmic systems. Importantly, this contribution concerns perceived transparency as measured in the present study and should not be interpreted as implying that greater objective disclosure is uniformly beneficial. The findings therefore support a more nuanced view, in which employees’ responses depend not only on the presence of algorithmic management but also on how such systems are understood and experienced.

Overall, the contribution of this study lies not in proposing an entirely new explanation of work withdrawal but in integrating research on algorithmic control with the stressor–strain–outcome logic of technostress theory and identifying AI anxiety and perceived algorithmic transparency as theoretically relevant psychological and contextual factors. In doing so, this study provides a more differentiated account of how algorithmic management is associated with employee withdrawal in digitally managed workplaces.

### 5.2 Practical implications

This study has certain practical implications for enterprises seeking to optimize algorithmic management practices. Given the cross-sectional design, these recommendations should be understood as theoretically informed implications rather than interventions directly validated by the present study.

First, when promoting algorithmic management, enterprises should avoid viewing it as a tool for improving efficiency and strengthening control. Algorithmic systems can support task allocation, process monitoring, performance evaluation and feedback management. However, employees may experience greater pressure, constraints, and uncertainty during this process. If enterprises focus only on the efficiency gains of algorithmic management while overlooking employees’ psychological experiences, such practices may lead to greater work withdrawal. Therefore, when applying algorithmic management, enterprises should consider both management efficiency and employee experiences.

Second, as algorithmic systems and artificial intelligence technologies continue to enter the workplace, employees may worry that their jobs will be replaced by technology and may also be concerned about their ability to understand or adapt to algorithmic rules. Such anxiety may be associated with greater psychological disengagement and work withdrawal. Therefore, when introducing algorithmic systems, enterprises should provide appropriate explanations, training, and support to help employees better understand the scope and role of algorithmic systems and address concerns related to technological replacement and adaptation.

Furthermore, enterprises should provide employees with understandable and useful information about the algorithmic management processes. Clear information about relevant rules, data sources, and evaluation criteria may make algorithmic procedures and work outcome evaluations more understandable to employees. However, greater information disclosure is not necessarily beneficial when the information is highly technical, incomplete, or difficult for employees to interpret. Therefore, organizations should emphasize meaningful transparency rather than disclosure alone.

In addition, enterprises should retain necessary interpersonal communication and human explanations in algorithmic management. Although algorithmic management systems can support the standardization and precision of management, they cannot completely replace the communication between managers and employees. In particular, when algorithmic decisions involve performance appraisal, work arrangements, compensation, or disciplinary decisions, enterprises should provide channels for explanation, appeal, and human review so that employees can understand the basis of these outcomes and have the opportunity to express their views. Such arrangements may help organizations retain the efficiency advantages of algorithmic management while providing greater opportunities for clarification, communication, and employee voice when misunderstandings, distrust, or difficulties in adapting to algorithmic systems arise.

The appropriate form of transparency and employee protection may also depend on the managerial function performed by an algorithmic system. For example, scheduling systems may require clear explanations of assignment rules and opportunities to correct inaccurate availability data, whereas monitoring and performance evaluation systems may require greater clarity regarding the data collected, evaluation criteria, and how employees can challenge questionable outcomes. When algorithm-supported decisions affect compensation, discipline, or other consequential employment outcomes, organizations should provide meaningful human reviews, mechanisms for contesting decisions, and clear responsibility for the final decision.

### 5.3 Research limitations and future prospects

This study had several limitations. First, the cross-sectional and self-report design does not establish temporal precedence or causal relationships and may be affected by reverse or reciprocal relationships, unmeasured confounding, common-source variance, recall bias, and social desirability. Participants were recruited through convenience and online channels, and the relatively young and well-educated composition of the sample, together with differences in respondents’ occupations, hierarchical levels, organizational contexts, and forms of algorithmic management, may limit the generalizability of these findings. Moreover, because the sample was restricted to employees from Internet-sector enterprises, the findings may not generalize fully to employees in other industries or organizational settings. Because work withdrawal behavior was self-reported, responses may also have been affected by respondents’ reluctance to disclose undesirable behaviors, such as lateness or unauthorized absence. Future research could combine self-reports with supervisor ratings or organizational records of withdrawal-related behavior.

Exposure to algorithmic management was also identified through respondents’ self-reports rather than independently verified organizational information. Because the eligibility assessment relied on respondents’ own understanding of the term “algorithmic management,” some variation in how participants interpreted the screening question may remain possible. The AI-anxiety measure captures relatively broad job-replacement and learning anxiety rather than anxiety toward a specific organizational algorithmic system, and only two dimensions of AI anxiety were included. Perceived algorithmic transparency also reflects employees’ subjective perceptions rather than objective system characteristics. In addition, the use of overall scores for constructs with multidimensional content may obscure potentially meaningful differences among their dimensions. The discriminant-validity results also indicated some empirical overlap between AI anxiety and work withdrawal behavior. Although the four-factor model provided better overall fit, the relatively high association between these constructs suggests that they may be difficult to distinguish fully in the present sample.

Finally, because this study was conducted in China, the findings may be culturally and institutionally specific. Future research could use longitudinal, experimental, or multisource designs, employ more context-specific and multidimensional measures, distinguish among different algorithmic-management functions, and replicate the model across different organizational and national contexts.

## Data Availability

The raw data supporting the conclusions of this article will be made available by the authors, without undue reservation.
