# Supplementary material for: Algorithmic management and employees’ work withdrawal behavior: the roles of AI anxiety and perceived algorithmic transparency
Source: Front Psychol. 2026 Oct 21;17:1916936. doi: 10.3389/fpsyg.2026.1916936 (PMC13730963; doi:10.3389/fpsyg.2026.1916936)
Supplement: Supplementary file 1 [file Supplementary_file_1.docx]

**Supplementary Appendix A**
**Full Questionnaire**
**Part A. Chinese Version Administered to Participants**

尊敬的受访者：

您好！感谢您参与本次问卷调查。本研究旨在了解员工对算法控制的感知及其对工作行为的影响，所得数据仅用于学术研究分析，不作任何商业用途。

本问卷采用匿名方式填写，不会收集您的姓名、联系方式、身份证号等可识别个人身份的信息。您所提供的答案将被严格保密，研究结果仅以整体统计形式呈现，不会涉及任何个人或具体单位的信息。

参与本问卷完全出于自愿，您可以根据自身真实感受作答，也可以在填写过程中随时退出。问卷中所有题项没有对错之分，您的回答不会对您的工作、评价或其他权益产生任何影响。

如果您继续填写本问卷，即表示您已了解上述说明，并自愿同意参与本研究。

再次感谢您的支持与配合！

1. 您所在的企业中是否会使用算法管理来监管您的日常工作 [单选题] *

○是

○否  (请跳至问卷末尾，提交答卷)

2. 您的性别是？[单选题] *

○男性

○女性

3. 您的年龄 [单选题] *

○25及以下

○26-30岁

○31-35岁

○36-40岁

○41-45岁

○46岁及以上

4. 您的受教育水平是 [单选题] *

○高中及以下

○大专

○本科

○研究生及以上

5. 您的工龄是 [单选题] *

○1年以下

○1-3年

○4-6年

○7-10年

○10年以上

6. 您的职位是 [单选题] *

○员工

○基层管理者

○中层管理者

○高层管理者

○从未

○很少

○一般

○经常

○总是

7. 您使用AI 的频率是[单选题] *

8. 本题目请选择选项3 [单选题] *

○选项1

○选项2

○选项3

○选项4

9. 以下题项旨在了解您对工作中AI/算法系统管理方式的感受，请根据您的实际工作体验选择最符合您情况的选项。[矩阵量表题] *

|  | 非常不同意 | 不同意 | 比较不同意 | 一般 | 比较同意 | 同意 | 非常同意 |
| --- | --- | --- | --- | --- | --- | --- | --- |
| 1.公司会用一套算法系统识别我的工作流程是否符合操作规范，并给出改进提示 | ○ | ○ | ○ | ○ | ○ | ○ | ○ |
| 2.公司会用一套算法系统实时监督我的工作状态 | ○ | ○ | ○ | ○ | ○ | ○ | ○ |
| 3.公司会用一套算法系统来实时追踪我的任务完成进度，并提供优化建议 | ○ | ○ | ○ | ○ | ○ | ○ | ○ |
| 4.我的每日工作任务由算法单方面指定且不可调整 | ○ | ○ | ○ | ○ | ○ | ○ | ○ |
| 5.公司会用一套算法系统根据我的能力数据安排适配的工作任务 | ○ | ○ | ○ | ○ | ○ | ○ | ○ |
| 6.公司会用一套算法系统来根据我的个人历史数据设定我在工作中达到的目标(生产力目标、时间目标、销售目标等) | ○ | ○ | ○ | ○ | ○ | ○ | ○ |
| 7.公司会用一套算法系统来制定我的周/月工作时间表 | ○ | ○ | ○ | ○ | ○ | ○ | ○ |
| 8.公司会用一套算法系统来决定我上下班打卡的时间 | ○ | ○ | ○ | ○ | ○ | ○ | ○ |
| 9.公司会用一套算法系统根据生产效率极值为我设定必须完成的时间目标 | ○ | ○ | ○ | ○ | ○ | ○ | ○ |
| 10.公司会用算法系统评估我的工作表现，并向我反馈能力成长轨迹 | ○ | ○ | ○ | ○ | ○ | ○ | ○ |
| 11.公司会用一套算法系统根据考勤记录与绩效评分计算我的工资收入 | ○ | ○ | ○ | ○ | ○ | ○ | ○ |
| 12.公司会用一套算法系统生成的未公开度量标准和计算指标来评估我的绩效 | ○ | ○ | ○ | ○ | ○ | ○ | ○ |

10. 湖北省的省会是 [单选题] *

○成都

○宜昌

○武汉

○南昌

11. 以下题项旨在了解您对人工智能（AI）在工作中应用的感受，请根据您的实际情况选择最符合您想法的选项。[矩阵量表题] *

|  | 非常不同意 | 不同意 | 一般 | 同意 | 非常同意 |
| --- | --- | --- | --- | --- | --- |
| 1.我担心在将来人工智能会取代我的工作 | ○ | ○ | ○ | ○ | ○ |
| 2.与比我聪明的人工智能一起工作，我感到焦虑 | ○ | ○ | ○ | ○ | ○ |
| 3.我担忧人工智能将取代多数人的工作 | ○ | ○ | ○ | ○ | ○ |
| 4.我不认为我能在人工智能的专业课上表现出色 | ○ | ○ | ○ | ○ | ○ |
| 5.理解人工智能算法需要很高的天赋和技能，这对我来说非常困难 | ○ | ○ | ○ | ○ | ○ |
| 6.人工智能技术更新变化快，对我来说学习难度很大 | ○ | ○ | ○ | ○ | ○ |

12. 以下题项旨在了解您对工作中AI/算法系统运行规则的了解程度，请根据您的实际情况，选择您对以下陈述的认同程度。[矩阵量表题] *

|  | 非常不同意 | 不同意 | 一般 | 同意 | 非常同意 |
| --- | --- | --- | --- | --- | --- |
| 1.我清楚地了解公司用于管理我工作的算法系统是如何运行的。 | ○ | ○ | ○ | ○ | ○ |
| 2.我可以查询公司用于管理我工作的算法系统的相关信息。 | ○ | ○ | ○ | ○ | ○ |
| 3.我能够较容易地获得有关公司算法管理系统运行方式的信息。 | ○ | ○ | ○ | ○ | ○ |

13. 请根据您最近一个月的实际工作情况，选择与您情况最符合的选项。[矩阵量表题] *

|  | 从不 | 很少 | 有时 | 时常 | 经常 |
| --- | --- | --- | --- | --- | --- |
| 1、在工作中产生过缺勤的念头。 | ○ | ○ | ○ | ○ | ○ |
| 2、在工作中出现神游、走神等情况 | ○ | ○ | ○ | ○ | ○ |
| 3、不愿意尽全力完成工作。 | ○ | ○ | ○ | ○ | ○ |
| 4、有离职的想法。 | ○ | ○ | ○ | ○ | ○ |
| 5、工作时间处理私人事务。 | ○ | ○ | ○ | ○ | ○ |
| 6、与同事讨论和工作无关的话题。 | ○ | ○ | ○ | ○ | ○ |
| 7、工作中存在擅自离开工作岗位的情况。 | ○ | ○ | ○ | ○ | ○ |
| 8、让他人代为完成自己的工作。 | ○ | ○ | ○ | ○ | ○ |
| 9、未经许可早退的情况。 | ○ | ○ | ○ | ○ | ○ |
| 10、耗用比规定更长的时间在午餐或休息上。 | ○ | ○ | ○ | ○ | ○ |
| 11、未经许可带走公司物品(原料或设备)。 | ○ | ○ | ○ | ○ | ○ |
| 12、工作时间打瞌睡的情况 | ○ | ○ | ○ | ○ | ○ |

**Supplementary Appendix A**

**Full Questionnaire**

*Part B. English Translation for Reference*

**Dear Respondent,**

Thank you for participating in this survey. This study aims to understand employees' perceptions of algorithmic control and its impact on work behavior. The data collected will be used solely for academic research and will not be used for any commercial purpose.

This questionnaire is completed anonymously. We will not collect your name, contact information, identification number, or other information that could identify you personally. Your responses will be kept strictly confidential. Research findings will be reported only in aggregate form and will not contain information about any individual or specific organization.

Participation in this survey is entirely voluntary. Please answer according to your actual experiences and feelings, and you may withdraw at any time while completing the questionnaire. There are no right or wrong answers. Your responses will not affect your work, evaluations, or any other rights or interests.

By continuing with this questionnaire, you indicate that you have read and understood the above information and voluntarily agree to participate in this study.

Thank you again for your support and cooperation.

**1. Does your organization use algorithmic management to supervise your daily work?** *[Single-choice item]*

- Yes
- No (Please skip to the end of the questionnaire and submit your response.)

**2. What is your gender?** *[Single-choice item]*

- Male
- Female

**3. What is your age?** *[Single-choice item]*

- 25 or below
- 26-30 years
- 31-35 years
- 36-40 years
- 41-45 years
- 46 years or above

**4. What is your educational level?** *[Single-choice item]*

- High school or below
- Junior college
- Bachelor's degree
- Graduate degree or above

**5. What is your tenure?** *[Single-choice item]*

- Less than 1 year
- 1-3 years
- 4-6 years
- 7-10 years
- More than 10 years

**6. What is your job level?** *[Single-choice item]*

- Employee
- Front-line supervisor
- Middle manager
- Senior manager

**7.How frequently do you use AI?** *[Single-choice item]*

- Never
- Rarely
- Sometimes
- Often
- Always

**8. For this item, please select Option 3.** *[Single-choice attention-check item]*

- Option 1
- Option 2
- Option 3
- Option 4

**9. The following items are intended to assess your perceptions of AI/algorithmic-system management in your work. Based on your actual work experience, please select the option that best describes your situation.** *[Matrix scale item]*

**Response scale:** Strongly disagree / disagree /Somewhat disagree / Neutral / Somewhat Agree /agree / Strongly agree

| **No.** | **Item** |
| --- | --- |
| 1 | The company uses an algorithmic system to identify whether my work process complies with operational standards and provides suggestions for improvement. |
| 2 | The company uses an algorithmic system to monitor my work status in real time. |
| 3 | The company uses an algorithmic system to track my task-completion progress in real time and provide suggestions for optimization. |
| 4 | My daily work tasks are unilaterally assigned by algorithms and cannot be adjusted. |
| 5 | The company uses an algorithmic system to assign work tasks that fit me based on data about my abilities. |
| 6 | The company uses an algorithmic system to set targets that I am expected to achieve at work based on my personal historical data (e.g., productivity targets, time targets, and sales targets). |
| 7 | The company uses an algorithmic system to schedule my weekly/monthly work timetable. |
| 8 | The company uses an algorithmic system to determine my clock-in and clock-out times. |
| 9 | The company uses an algorithmic system to set mandatory time targets for me based on maximum production efficiency. |
| 10 | The company uses an algorithmic system to assess my work performance and provide feedback on the trajectory of my capability development. |
| 11 | The company uses an algorithmic system to calculate my wage income based on attendance records and performance ratings. |
| 12 | The company evaluates my performance using undisclosed measurement standards and computational indicators generated by an algorithmic system. |

**10. What is the capital city of Hubei Province?** *[Single-choice factual-check item]*

- Chengdu
- Yichang
- Wuhan
- Nanchang

**11. The following items are intended to assess your feelings about the use of artificial intelligence (AI) at work. Based on your actual situation, please select the option that best reflects your view.** *[Matrix scale item]*

**Response scale:** Strongly disagree / Disagree / Neutral / Agree / Strongly agree

| **No.** | **Item** |
| --- | --- |
| 1 | I am worried that artificial intelligence will replace my job in the future. |
| 2 | I feel anxious about working with artificial intelligence that is smarter than I am. |
| 3 | I am worried that artificial intelligence will replace most people's jobs. |
| 4 | I do not think I would perform well in a course specializing in artificial intelligence. |
| 5 | Understanding artificial intelligence algorithms requires a high level of talent and skills, which is very difficult for me. |
| 6 | Artificial intelligence technology changes and updates rapidly, which makes it very difficult for me to learn. |

**12. The following items are intended to assess your understanding of the operating rules of AI/algorithmic systems used in your work. Based on your actual situation, please indicate the extent to which you agree with each statement.** *[Matrix scale item]*

**Response scale:** Strongly disagree / Disagree / Neutral / agree / Strongly agree

| **No.** | **Item** |
| --- | --- |
| 1 | I clearly understand how the algorithmic system used by the company to manage my work operates. |
| 2 | I can access information about the algorithmic system used by the company to manage my work. |
| 3 | I can relatively easily obtain information about how the company's algorithmic management system operates. |

**13. Based on your actual work situation during the past month, please select the option that best describes your situation.** *[Matrix scale item]*

**Response scale:** Never / Rarely / Sometimes / Quite often / Frequently

| **No.** | **Item** |
| --- | --- |
| 1 | I have had thoughts of being absent from work. |
| 2 | I have experienced mind-wandering or being distracted at work. |
| 3 | I have been unwilling to make every effort to complete my work. |
| 4 | I have thought about leaving my job. |
| 5 | I have handled personal matters during work hours. |
| 6 | I have discussed topics unrelated to work with colleagues. |
| 7 | I have left my workplace without permission. |
| 8 | I have had someone else complete my work for me. |
| 9 | I have left work early without permission. |
| 10 | I have spent longer than permitted on lunch or breaks. |
| 11 | I have taken company property (raw materials or equipment) without permission. |
| 12 | I have dozed off during work hours. |

Supplementary Table S1. Item-Level Descriptive Statistics and Response Frequencies for the Work Withdrawal Behavior Scale

| **Item** | **M** | **SD** | **Skewness** | **1 = Never (%)** | **2 = Rarely (%)** | **3 = Sometimes (%)** | **4 = Quite often (%)** | **5 = Frequently (%)** |
| --- | --- | --- | --- | --- | --- | --- | --- | --- |
| WWB1 | 2.91 | 0.713 | 0.031 | 1.4 | 24.9 | 55.5 | 17.3 | 0.9 |
| WWB2 | 3.35 | 0.703 | −0.149 | 0.6 | 8.4 | 50.0 | 37.9 | 3.2 |
| WWB3 | 2.72 | 0.758 | 0.082 | 4.0 | 33.8 | 49.4 | 11.8 | 0.9 |
| WWB4 | 2.73 | 0.738 | 0.125 | 3.2 | 33.8 | 50.6 | 11.6 | 0.9 |
| WWB5 | 3.10 | 0.684 | 0.033 | 0.3 | 16.8 | 56.6 | 25.1 | 1.2 |
| WWB6 | 2.97 | 0.833 | 0.156 | 2.0 | 27.5 | 45.4 | 22.3 | 2.9 |
| WWB7 | 2.95 | 0.748 | 0.210 | 1.2 | 25.1 | 53.5 | 18.2 | 2.0 |
| WWB8 | 2.69 | 0.690 | −0.032 | 2.9 | 35.5 | 51.4 | 10.1 | 0.0 |
| WWB9 | 3.13 | 0.682 | −0.165 | 0.9 | 14.2 | 57.2 | 26.9 | 0.9 |
| WWB10 | 3.11 | 0.678 | 0.251 | 0.3 | 14.7 | 60.7 | 22.0 | 2.3 |
| WWB11 | 2.95 | 0.727 | 0.075 | 1.2 | 24.3 | 54.0 | 19.4 | 1.2 |
| WWB12 | 3.17 | 0.733 | 0.116 | 0.6 | 14.7 | 54.6 | 26.9 | 3.2 |
